# Determinants of consumption-day amounts applicable for the estimation of usual dietary intake with a short 24-h food list

**DOI:** 10.1017/jns.2016.26

**Published:** 2016-08-15

**Authors:** Johanna Freese, Mihaela Pricop-Jeckstadt, Thorsten Heuer, Matthias Clemens, Heiner Boeing, Sven Knüppel, Ute Nöthlings

**Affiliations:** 1 Department of Nutrition and Food Sciences, Nutritional Epidemiology, University of Bonn, Endenicher Allee 11-13, 53115 Bonn, Germany; 2 Institute for Medical Informatics and Biometry, TU-Dresden, Fetscherstr. 74, 01307 Dresden, Germany; 3 Department of Nutritional Behaviour, Max Rubner-Institut, Federal Research Institute of Nutrition and Food, Haid-und-Neu-Str. 9, 76131 Karlsruhe, Germany; 4 Department of Epidemiology, German Institute of Human Nutrition Potsdam-Rehbruecke, Arthur-Scheunert-Allee 114-116, 14558 Nuthetal, Germany

**Keywords:** Dietary assessment, Nutritional epidemiology, Large-scale settings, Usual dietary intake, Statistical modelling, 24HDR, 24-h dietary recall, NVS II, German National Nutrition Survey II

## Abstract

Next to the information on frequency of food consumption, information on consumption-day amounts is important to estimate usual dietary intake in epidemiological studies. Our objective was to identify determinants of consumption-day amounts to derive person-specific standard consumption-day amounts applicable for the estimation of usual dietary intake using separate sources to assesss information on consumption probability and amount consumed. 24-h Dietary recall data from the German National Nutrition Survey II (*n* = 8522; aged 20–80 years) conducted between 2005 and 2007 were analysed for determinants of consumption-day amounts of thirty-eight food and beverage groups using LASSO variable selection for linear mixed-effects models. Determinants included sex, age, BMI, smoking status, years of education, household net income, living status and employment status. Most often, sex, age and smoking status were selected as predictors for consumption-day amounts across thirty-eight food groups. In contrast, living with a partner, employment status and household net income were less frequently chosen. Overall, different determinants were of relevance for different food groups. The number of selected determinants ranged from eight for coffee and juice to zero for cabbage, tea, root vegetables, leafy vegetables, fruit vegetables, legumes, offal, vegetable oils, and other fats. For the estimation of usual dietary intake in a combined approach with a 24-h food list, person-specific standard consumption-day amounts could be used. Sex, age and smoking status were shown to be the most relevant predictors in our analysis. Their impact on the estimation of usual dietary intake needs to be evaluated in future studies.

It has been suggested that approaches of combining instruments, e.g. repeated 24-h dietary recall (24HDR) with a FFQ, may provide high-quality dietary information, especially for the assessment of foods that are not consumed every day^(^^1^^–^^5^^)^. These methods presume that the usual dietary intake of a subject equals the consumption probability of a food on a given day multiplied by the average amount of intake of that food on a typical consumption day. In theory, the information on consumption probability and consumption-day amount could be derived from different instruments. It has been previously noted that most of the variation in food consumption is explained by frequency of intake^(^^6^^,^^7^^)^. We therefore recently proposed the application of a short 24-h food list for the estimation of consumption probability, which only enquires about whether or not a food was consumed on the previous day^(^^8^^)^. Individual information on amounts consumed is replaced by standard consumption-day amounts. The 24-h food list is intended to be used in a combined approach with an FFQ to estimate usual dietary intake. This approach is currently implemented in a large German cohort study, the German National Cohort^(^^9^^,^^10^^)^, and reflects an effort to reduce demands on time in dietary assessment in large-scale settings.

Information on standard consumption-day amounts of foods may be derived from different sources: representative national dietary survey data or detailed dietary assessments on a subgroup of the population under study are two examples. Furthermore, standard consumption-day amounts can either be assigned identically for all study participants, or specifically stratified for subgroups of the study population such as men and women^(^^7^^)^, or estimated by using appropriate statistical models^(^^1^^,^^3^^)^.

Thus far, there has been some research on factors influencing both the amount of foods consumed and frequency of consumption. It has been shown that amounts consumed differed by sex, age or BMI^(^^7^^,^^11^^)^. According to previous research in surveys, sociodemographic factors such as education^(^^12^^)^, income^(^^13^^)^, family status^(^^14^^)^, socio-economic status^(^^15^^)^ and smoking status^(^^16^^,^^17^^)^ were associated with food and beverage consumption. However, these studies looked at the factors separately, so that it remains unclear whether or not the factors have a joint impact on the individual consumption-day amounts. Moreover, different factors may be of relevance for amounts of different groups of foods and beverages.

Implementing person-specific standard consumption-day amounts, also differently across food groups, may lead to more precise estimates of individual usual dietary product intake. Therefore, the aim of the present study was to identify determinants explaining variation in consumption-day amounts across different food groups using national dietary survey data.

## Subjects and methods

### Survey methodologies and study population

The German National Nutrition Survey II (NVS II) was carried out from November 2005 to January 2007 in a representative sample of German-speaking residents^(^^18^^)^. Amongst others, dietary intake in the NVS II was assessed on two non-consecutive days using EPIC-Soft (renamed GloboDiet in 2014), a well-established computerised 24HDR, conducted by trained interviewers over the telephone^(^^19^^,^^20^^)^. The two 24HDR were randomly sampled and approximately proportionally distributed over weekdays and weekends (75 and 25 %, respectively)^(^^20^^)^. The interviews were open-ended and included probing questions and integrated quality checks. In detail, participants were asked to recall the foods and drinks they had consumed the previous day. A quick list was used to report all foods and beverages consumed. This was followed by a detailed enquiry on food preparation, portion size, brand names or additions^(^^19^^,^^21^^)^. Portion size was assessed using household measures, standard units and a picture booklet^(^^20^^)^. In the end, additional data checks were conducted by the research team to identify and correct for data input errors (e.g. for large and small quantities, extremes in the calculated energy and macronutrient intake).

In total, 12 502 participants of the NVS II aged 20–80 years completed two EPIC-Soft 24HDR. For the present study, NVS II participants were excluded if they were breastfeeding or pregnant women, or if they had a particular diet such as a slimming diet or a diet related to health conditions (*n* = 2672). It was assumed that those study participants did not consume typical amounts of foods and beverages. In addition, NVS II participants with missing values on socio-economic factors and smoking status were excluded (*n* = 1308). This resulted in an analytical study population of 8522 participants.

Demographic, socio-economic and lifestyle variables were assessed in a computer-assisted personal interview^(^^18^^)^. Years of education were determined according to the International Standard Classification of Education 1997^(^^22^^)^. Anthropometric measures were assessed in three different ways: (1) measures of weight and height following a standardised protocol^(^^23^^)^ (*n* = 5809); (2) self-reported weight and height (*n* = 2694); and (3) for participants with missing information on weight and height, the BMI was calculated based on sex- and age-specific mean values for BMI from NVS II participants with information on measured or self-reported weight and height (*n* = 19).

### Classification of food groups

Foods were grouped according to a newly developed tool, the short 24-h food list^(^^8^^)^. Food and drink items were categorised into thirty-eight groups of food and beverages. The food groups and the general food items within each group are listed in Supplementary Table S1. For each food group, the consumption-day amount, defined as the amount consumed in g per d, was calculated.

### Statistical analysis

Descriptive statistics of the study population are presented as percentages for categorical variables and as mean and standard deviation for continuous variables.

To check for multicollinearity between determinants, the variance inflation factor was calculated. The φ coefficient was determined to measure the association between predictors for consumption-day amounts. The analyses were performed using the statistical software package SAS (version 9.4, 2008; SAS Institute Inc.).

The consumption-day amount (g/d) was treated as the dependent variable. Sex, age (years), BMI (kg/m^2^), smoking status (current; former; never), years of education (9–10; 12–13; 14–16; 17–18 years), living together with a partner (yes; no), household net income (<1500 €; 1500 € to <3000 €; ≥3000 €) and employment status (yes; no) were investigated as determinants.

To determine the most relevant predictors of consumption-day amounts, the LASSO, as a popular shrinkage and variable selection method for linear mixed-effects models, was used (see Supplementary Methods for detailed information on the statistics). The dependent variable was Box-Cox transformed to obtain normally distributed residuals^(^^24^^)^. Further statistical analysis was conducted using the package lmmlasso in R (version 3.1.1). To pick the most suitable LASSO model, the Bayesian information criterion was used. The Bayesian information criterion is advocated in the original article proposing the LASSO for linear mixed-effects models based on the authors’ empirical experience^(^^25^^)^.

## Results

The analytical sample comprised 3989 men (47 %) and 4533 women (53 %) (Table 1). Overall, the mean age of the study population was 48 years and the mean BMI was 26·0 kg/m^2^.

**Table 1.** Characteristics of participants of the German National Nutrition Survey II (*n* = 8522) (Percentages, mean values and standard deviations)

	Age group (years)		
	20–34	35–64	65–80
*n*	1526	5603	1393
Female (%)	54·9	53·6	49·6
Age (years)			
Mean	27·6	48·0	70·0
sd	4·4	8·3	4·2
BMI (kg/m^2^)			
Mean	24·2	26·1	27·3
sd	4·5	4·4	4·1
Smoking status (%)			
Never	52·7	45·1	61·4
Former	11·9	25·5	28·7
Current	35·5	29·3	9·9
Years of education (%)*			
9–10 years	5·9	4·8	17·1
12–13 years	53·5	50·4	48·0
14–16 years	22·2	21·0	16·4
17–18 years	18·4	23·8	18·5
Household net income (%)			
<1500 €	30·4	17·3	32·0
1500 to <3000 €	47·9	49·4	52·9
≥3000 €	21·7	33·4	15·1
Living together with a partner (%)	57·0	82·9	76·9
Employed (%)	78·0	76·9	5·9

* According to the International Standard Classification of Education 1997^(^^22^^)^.

The results of the LASSO variable selection explaining the consumption-day amounts across the thirty-eight food and beverage groups, using the Bayesian information criterion as the selection criterion, are shown in Tables 2 and 3. Overall, sex was selected as the predictor most often (*n* = 26) followed by age (*n* = 21) and smoking status (*n* = 18) (Table 2). In contrast, employment status, living with a partner and household net income were chosen less frequently. Years of education and BMI were selected for the models of thirteen and twelve food groups, respectively. With respect to single food groups, all eight determinants were of relevance for the consumption-day amount of coffee and juice (Table 3). In contrast, no predictor was selected for the models of the food groups cabbage, legumes, offal, tea, fruit vegetables, leafy vegetables, root vegetables, vegetable oils and other fats. For cake, potatoes and poultry, sex was the only determinant selected, whereas for the food group other vegetables, solely age was chosen.

**Table 2.** Relevance of determinants for consumption-day amounts across thirty-eight food groups using LASSO variable selection*

Determinant	Food groups with positive selection (*n*)
Sex	26
Age	21
Smoking status	18
Years of education	14
BMI	12
Employment	10
Living with a partner	9
Household net income	8

* Selection criterion: Bayesian information criterion.

**Table 3.** Selected determinants for consumption-day amounts across thirty-eight food and beverage groups*

Food group†	Sex	Age	Smoking status	Years of education	BMI	Employment	Living with a partner	Household net income
Coffee	x	x	x	x	x	x	x	x
Juice	x	x	x	x	x	x	x	x
Processed meat	x	x	x	x	x	x		x
Other non-alcoholic drinks	x	x	x	x	x	x	x	
Bread	x		x		x	x	x	x
Milk and dairy products	x	x	x	x		x	x	
Wine	x	x	x		x	x		x
Beer	x	x	x		x		x	x
Cheese	x	x		x		x		x
Butter	x	x	x	x	x			x
Other alcoholic drinks	x	x	x	x	x			
Nuts	x	x	x	x		x		
Eggs	x	x	x				x	
Meat	x	x	x		x			
Cereals	x	x	x				x	
Soft drinks	x	x	x	x				
Sugar	x	x			x		x	
Fresh fruits		x	x	x				
Sauces	x	x	x					
Pasta/rice	x	x			x			
Soup	x			x				
Other fruits	x			x				
Fish	x			x				
Spirits		x	x					
Margarine	x					x		
Cake	x							
Potatoes	x							
Other vegetables		x						
Poultry	x							

* LASSO variable selection for linear-effect models was used; selection criterion: Bayesian information criterion.

† Food groups cabbage, tea, root vegetables, leafy vegetables, fruit vegetables, legumes, offal, vegetable oils and other fats are not shown because no determinant was selected for those food groups.

Both the variance inflation factor and the φ coefficient indicated a correlation only of the determinants years of education and household net income in the models (variance inflation factor >2; *r* φ = 0·37) (data not shown).

## Discussion

The findings of the present study indicate that all demographic and socio-economic factors investigated were relevant for the consumption-day amount of specific food groups. However, differences in importance across food and beverage groups were observed. Sex was shown to be the most relevant predictor followed by age and smoking status. In contrast, household net income was less important. Interestingly, BMI was overall less relevant for consumption-day amount of food groups than expected.

The estimation of usual dietary intake in large-scale prospective studies is a current topic of interest. It has been suggested that an approach of combining instruments such as repeated 24HDR and FFQ may provide high-quality dietary information, especially for the assessment of foods that are not consumed every day^(^^1^^–^^5^^)^. These methods presume that the usual dietary intake of a subject equals the consumption probability of a food on a given day multiplied by the average amount of intake of that food on a typical consumption day. Although multiple administrations of detailed 24HDR would be optimal, this is impracticable in large-scale cohort studies due to the associated high costs and time expenditure^(^^26^^)^. Detailed web-based 24HDR developed for self-administered use are likely to be more cost-effective with respect to administration^(^^27^^)^, but might still be time-consuming for the study participant, and are only available in the UK^(^^28^^)^ and the USA^(^^29^^)^ so far. For Germany, we recently proposed a short 24-h food list, which only enquires about whether or not a food was consumed on the previous day^(^^8^^)^. Instead of individual amounts consumed, standard consumption-day amounts can be applied for the estimation of usual dietary intake.

Our study indicated that different determinants were of importance for different food groups. For some food groups, six to eight predictors were important whereas for other groups, none was chosen. Overall, sex, age and smoking status were shown to be mostly relevant, but for some food groups further determinants carried information. Thus, if available, person-specific consumption-day amounts could be predicted differently across foods. However, the gain in information for the estimation of usual dietary intake might have to be evaluated in future research.

Information on standard consumption-day amounts may be derived from different sources of data. First, national dietary survey data such as the NVS II in Germany can be used^(^^18^^)^. Second, the application of 24HDR on a subgroup of the population under study can be conducted. National survey data might provide representative data of the population of a country. Moreover, survey sample sizes are typically large and, thus, the variety in the pictured diet might be high. This may lead to more reliable data for the computation of standard consumption-day amounts. In contrast, the application of 24HDR in a subgroup of the population under study might be preferable for specific ethnic populations.

To take into account covariates predicting consumption-day amounts, two general strategies are possible. First, standard consumption-day amounts could be derived using stratified mean or median intakes of the respective food items as it has been done previously^(^^7^^,^^30^^)^; and second, amounts could be estimated by using appropriate statistical models^(^^1^^,^^3^^,^^31^^)^. Tooze *et al.*^(^^1^^)^ stated that using statistical models may result in a more efficient estimation than does stratification. Especially when applying a number of person-specific factors, as is suggested by the present findings, stratification may lead to very small samples for specific strata combinations. Therefore, the application of prediction models seems to be advantageous for the proposed approach.

Of note, person-specific standard consumption-day amounts can only be used for determinants which actually have been measured in the respective study. All of the proposed determinants are nowadays typically assessed in nutritional epidemiological studies meaning that no additional assessment effort would be necessary^(^^32^^,^^33^^)^. However, for household net income, which was one of the factors proposed to be important for consumption-day amounts, no general standards are available for the generation of categories. Even if prediction models are applied instead of stratification, this variable needs to be categorised for analysis. Thus, future research should evaluate the application of different strata according to the estimation of usual dietary intake. A correlation between the variables household net income and years of education was observed. Thus, the use of one of the variables would be sufficient for the application of person-specific standard consumption-day amounts.

Our study has some limitations. First, to identify determinants that explain variation in consumption-day amounts of foods and beverages, food items of different serving size were combined into commonly used food groups, reducing some of the variation in the data. Second, although a broad range of possibly informative socio-economic and anthropometric factors was considered, there may be other important determinants such as physical activity that additionally influence the amount consumed. It was, however, not possible to include this variable in our analysis since information on physical activity in the NVS II was assessed for a subgroup of participants only. The remaining study population would have weakened the analysis. Third, the application of person-specific standard consumption-day amounts is limited by sufficient variation of determinants in a study population.

The fact that representative contemporary German dietary survey data were used to analyse determinants of consumption-day amounts can be considered a strength. Thus, the results are suitable for use all over Germany. State-of-the-art variable selection procedures were applied to analyse the relevance of consumption-day amount predictors. The application of person-specific standard consumption-day amounts may lead to more precise estimates of usual dietary intake in large-scale settings that are limited by the costs and logistics of data collection.

### Conclusion

For the estimation of usual dietary intake in combined approaches, person-specific standard consumption-day amounts could be used. Sex, age and smoking status were shown to be the most relevant determinants in our analysis. However, for some food groups further determinants were explanatory, suggesting, if possible, to predict consumption-day amounts based on the whole set of relevant variables. The impact of person-specific standard consumption-day amounts for the estimation of intake of single food items might need to be evaluated in future research.

## Supplementary material

For supplementary material accompanying this paper visit http://dx.doi.org/10.1017/jns.2016.26. click here to view supplementary material
