# Supplementary material for: Determinants of consumption-day amounts applicable for the estimation of usual dietary intake with a short 24-h food list
Source: J Nutr Sci. 2016 Aug 15;5:e35. doi: 10.1017/jns.2016.26 (PMC5048183; doi:10.1017/jns.2016.26)
Supplement: Supplementary file 1 [file S2048679016000264sup.zip › S2048679016000264sup002.doc]

**Online Supporting Material**

**Supplementary Methods**

Let be the true food intake and be the 24-hour recall measurement of dietary intake for an individual on day. The true food intake for an individual is defined as the mean of repeated single-day intakes. Since the dietary data distribution is skewed, it requires a Box-Cox transformation of that leads to the transformed 24-hour recall with α as Box-Cox transformation factor .

We assume that the transformed 24-hour recall is an unbiased estimator of transformed usual food intake, as it is customary in the literature (Assumption A in Dodd et al. ). Hence, the corresponding linear mixed model can be written with the notations from Schelldorfer et al. as where is the grouping index, is the observation index within a group corresponding to subject, is the vector of transformed 24-hour recalls, is the fixed-effects design matrix, is the vector of fixed effects, is the random-effects design matrix and is the the subject-specific vector of random effects . The following conditions are considered fulfilled:

i. the vector of errors are uncorrelated for 𝑖=1,…,𝑁.
ii. are uncorrelated for.
iii. are independent.

In our case, takes the values 1 or 2, differs from food group to food group, and we have an unbalanced one-way ANOVA design.

Lasso is a very popular statistical approach to variable selection based on the size of the fixed effects. In the setting of linear mixed models, this was first applied in Schelldorfer et al. where some theoretical properties were also studied . Instead of minimizing the maximum likelihood function (MLF), the lasso objective function adds a penalization term to MLF. This leads to a sparse estimator whose components are equal to zero for small effects. The amount of penalization is controlled by a tuning parameter that plays a decisive part in the quality of the estimator (i.e. the number of zeros and its convergence properties).

The authors of the original article recommend the Bayesian Information Criterion due to the better performance in numerical examples. This choice is already programmed in the lmmlasso R-package and was also used in our applications. Therefore, the grid for the-values was chosen such that it covers enough values for all food groups to obtain the U-form of the BIC graphical representation.

**Supplementary References**

1.	Box GEP, Cox DR (1964) An Analysis of Transformations. *J Roy Stat Soc B* **26**, 211-252.

2.	Dodd KW, Guenther PM, Freedman LS*, et al.* (2006) Statistical methods for estimating usual intake of nutrients and foods: a review of the theory. *J Am Diet Assoc* **106**, 1640-1650.

3.	Schelldorfer J, Buhlmann P, Van De Geer S (2011) Estimation for High-Dimensional Linear Mixed-Effects Models Using l(1)-Penalization. *Scand J Stat* **38**, 197-214.
