# Supplementary material for: Determinants of consumption-day amounts applicable for the estimation of usual dietary intake with a short 24-h food list
Source: J Nutr Sci. 2016 Aug 15;5:e35. doi: 10.1017/jns.2016.26 (PMC5048183; doi:10.1017/jns.2016.26)
Supplement: Supplementary file 1 [file S2048679016000264sup.zip › S2048679016000264sup001.doc]

**Online Supporting Material**

Supplementary Table S1. Assignment of food items to groups of foods and beverages

| *Food or beverage group* | *Included food items* |
| --- | --- |
| Potatoes | Potatoes, pan-fried potatoes, mashed potatoes, potato dumplings, potato salad, filled potatoes, potato pancake, chips/French fries, other |
| Leafy vegetables | Lettuce, spinach |
| Fruit vegetables | Capsicum/pepper, tomatoes, cucumber, pickled cucumber/gherkin, zucchini/courgette, aubergine/eggplant, tomato sauce |
| Root vegetables | Carrots |
| Cabbage | Turnip cabbage (kohlrabi), broccoli, cauliflower, white cabbage, kale/borecole, Brussels sprouts, red cabbage, sauerkraut |
| Other vegetables | Olives, avocado, mushrooms, asparagus, mixed vegetables, onions, garlic, other |
| Legumes | Legumes |
| Fresh fruits | Apple, pear, orange, tangerine, kiwi, cherries, plum, mirabelle plum, peach, apricot, nectarine, pomegranate, grapes, melon, banana, strawberries, blueberries, raspberries, gooseberries, fresh fig, physalis/cape gooseberry, pineapple, fruit salad, other |
| Other fruits | Stewed fruit/canned fruit, dried fruit |
| Nuts | Nuts, seeds, flax seed, trail mix, other |
| Milk and dairy products | Milk, soured milk, buttermilk, hot/cold cocoa, flavoured milk (drinks), (whipped) cream, kefir, plain yoghurt, flavoured yoghurt, pudding, crème fraiche, plain curd (quark), curd with herbs, quark dessert, spread, other |
| Cheese | Cream cheese/cottages cheese, soft cheese, mozzarella, feta, semi-hard and hard cheese, sour milk cheese, other |
| Pasta/rice | Pasta/noodles, whole grain pasta, rice, groats, other grains |
| Bread | Brown (rye wheat) bread and buns, multigrain bread and buns, rye bread and buns, spelt bread and buns, whole grain bread and buns, whole grain toast, toast, croissant, wheat bread and buns/baguette, lye pretzel/breads, flatbread, other |
| Other cereals | Muesli, corn flakes, wholemeal, rolled cereals/oat flakes, puffed rice, other cereals, crisps and crackers |
| *Continued on the following page* | |

Supplementary Table S1. Assignment of food items to groups of foods and beverages *(continued)*

| *Food or beverage group* | *Included food items* |
| --- | --- |
| Meat | Beef, veal, pork, lamb, venison, mixed ground meat, German beef roulade, beef goulash, roast pork, pork goulash, gyros, shashlik/meat skewer, hamburger/meatball |
| Poultry | Poultry, chicken/turkey ragout |
| Processed meat | Liverwurst, salami, mettwurst, cabanossi, bologna/polony, ham sausage, cooked ham, raw ham, poultry sausage, aspic, collared pork, blood sausage, frankfurter/wiener/hot dog, bratwurst, Bavarian veal sausage, Bavarian meat loaf, meat salad, other |
| Offals | Liver, other offals |
| Fish | Salmon, mackerel, herring, herring salad, salted herring, fried herring, rolled pickled herring, hot smoked herring, sprat, eel, redfish, trout, tuna, saithe/pollock, codfish, fish sticks, fish bake, calamari, craps/shellfish, other |
| Eggs | Egg salad, boiled egg, fried/scrambled eggs, other |
| Vegetable oils | Olive oil, vegetable oil, wheat germ oil, safflower oil, sunflower oil, grape seed oil, linseed oil, rapeseed oil, other |
| Butter | Butter |
| Margarine | Margarine |
| Other fats | Dripping/lard, bacon |
| Sugar | Honey, jam, sugar beet molasses, hazelnut spread, chocolate mousse, tiramisu, cold sweet soup with fruits, red fruit jelly, ice cream, other dessert, filled chocolates, chocolate bar, other chocolate or sweets with chocolate, other sweets without chocolate, sugar as addition |
| Cake | Yeast cake and pastry, tart/pie, cream pie/cake with butter crème or custard filling, cheesecake, pound cake/muffins, cookies or biscuits with chocolate icing, cookies or biscuits without chocolate icing, pancake, waffles, other |
| Juice | Multi-vitamin juice, apple juice, orange juice, grape juice, grapefruit juice, elder juice, lemon juice, other fruit juice, tomato juice, other vegetable juice |
| Soft drinks | Lemonade, diet lemonade, cola, diet cola, non-alcoholic beer, malt beer, other |
| *Continued on the following page* | |

Supplementary Table S1. Assignment of food items to groups of foods and beverages (*continued*)

| *Food or beverage group* | *Included food items* |
| --- | --- |
| Other non-alcoholic beverages | Mineral water/drinking water, herbal tea, fruit tea |
| Coffee | Coffee/espresso, coffee/espresso with (concentrated) milk, coffee without caffeine, coffee without caffeine with (concentrated) milk, cappuccino/caffè latte |
| Tea | Black tea, black tea with (concentrated) milk, green tea, other hot drinks |
| Wine | White wine, red wine, rosé wine |
| Beer | Beer, strong beer/malt liquor |
| Spirits | Spirits |
| Other alcoholic beverages | Sparkling wine, beer shandy, liqueur, hot wine punch, wine spritzer, other mixed beverages, other |
| Sauces | Gravy with vegetables, gravy with meat/fish, gravy with side dish, salad dressing, mayonnaise, ketchup, other |
| Soup | Vegetable stew, stew with meat, clear soup, crème of vegetable soup, other |
